# Quantum Mechanical Engine for the Quantum Rabi Model

**DOI:** 10.3390/e20100767

**Published:** 2018-10-07

**Authors:** Gabriel Alvarado Barrios, Francisco J. Peña, Francisco Albarrán-Arriagada, Patricio Vargas, Juan Carlos Retamal

**Affiliations:** 1 Departamento de Física, Universidad de Santiago de Chile (USACH), Avenida Ecuador 3493, Santiago 9170124, Chile; 2 Departamento de Física, Universidad Técnica Federico Santa María Casilla 110V, Valparaíso 2340000, Chile; 3 Center for the Development of Nanoscience and Nanotechnology, Estación Central, Santiago 9170124, Chile

**Keywords:** quantum thermodynamics, quantum Rabi model, isoenergetic cycle

## Abstract

We consider a purely mechanical quantum cycle comprised of adiabatic and isoenergetic processes. In the latter, the system interacts with an energy bath keeping constant the expectation value of the Hamiltonian. In this work, we study the performance of the quantum cycle for a system described by the quantum Rabi model for the case of controlling the coupling strength parameter, the resonator frequency, and the two-level system frequency. For the cases of controlling either the coupling strength parameter or the resonator frequency, we find that it is possible to closely approach to maximal unit efficiency when the parameter is sufficiently increased in the first adiabatic stage. In addition, for the first two cases the maximal work extracted is obtained at parameter values corresponding to high efficiency, which constitutes an improvement over current proposals of this cycle.

## 1. Introduction

The possibility to create nano-scale devices which are more efficient than current classical counterparts motivates the study of the quantum version of the very well known cycles of classical thermodynamics [1,2,3,4,5,6]. The interest in these concepts has derived in the experimental construction of some quantum thermal machines [7,8,9]. The main hypothesis is that there is a relation between the quantum nature of the working substance and the first law of thermodynamics, this link has been already established by others [10].

In 2000, Bender et al. proposed a thermodynamical cycle with no classical analogue [11], which involved the replacement of the heat baths for so-called “energy baths”. This was originally presented as a proposal for the substitution of the concept of temperature with the expectation value of the system Hamiltonian [11,12]. When the system is coupled to an energy bath it evolves through an isoenergetic process, during which the expectation value of the Hamiltonian is constant. This cycle has been mostly considered for a single non-relativistic confined particle [13,14,15,16,17,18,19,20], and its optimization has also been a focus of study [21,22,23]. Recently, it was extended to the case of relativistic regime by considering the single-particle Dirac spectrum [24,25] and has also been extended to multilevel systems [26,27].

On the other hand, light-matter systems are described in the more basic sense by the quantum Rabi model [28]. This model describes the interaction of a single electromagnetic mode with a two-level system (TLS), and it has been studied in a wide range of the coupling parameter [29,30,31]. In particular, the ultrastrong-coupling (USC) regime, which has been experimentally realized [30], corresponds to the case where the coupling strength and the resonator frequency become comparable. The light-matter interaction in the USC regime presents interesting properties, such as parity symmetry, and anharmonic energy spectrum [32]. These properties have led to remarkable applications of systems described by the USC, also termed quantum Rabi systems (QRS), such as fast quantum gates [33], efficient energy transfer [34,35], and generation of non-classical states [36,37]. Further, current progress in superconducting circuit technology has enabled the manipulation of several parameters of QRSs [38,39,40,41,42,43,44,45,46]. These results have motivated the study of this system as a working susbtance of quantum thermodynamical cycles [5,6]. This progress, together with the anharmonicity and nonlinearity spectrum of the QRS, constitutes an interesting system to investigate the performance of this quantum cycle involving isoenergetic processes.

In this work we study a quantum cycle comprising adiabatic and isoenergetic processes, where the working substance corresponds to a two-level system interacting with a single electromagnetic mode described by the quantum Rabi model. Notice that this choice involves a different physical scenario from other thermodynamical cycles, such as quantum Otto cycle [5,6], since here the system does not interact with thermal reservoirs. This means that the total work extracted and the efficiency will be completely different from that of Reference [5] even under the variation of the same parameters. In the case of the present work, the efficiency is not bounded by Carnot’s efficiency. Here, we consider an analytical approximation of the energy levels which allows for qualitative and quantitative description of the thermodynamical quantities depending on the range of validity of the approximation. We obtain the total work extracted and efficiency of the cycle for the variation of each one of the parameters of the model, namely, the coupling strength, the resonator frequency, and the two-level system frequency. For the cases where the energy spectrum shows nonlinearity and degeneracy, we see that the cycle performance is improved. In particular, we find that the nonlinear dependence of the energy levels on either the coupling strength, *g*, or the resonator frequency, ω, allows for the cycle efficiency to closely approach to maximal unit value, when the parameter is sufficiently increased in the first adiabatic stage.

### 1.1. Quantum Rabi Model

We will consider a working substance composed of a light-matter system described by the quantum Rabi model [28,32], which reads as:

(1) $$H=\hbar \Omega \sigma_{z}+\hbar \omega a^{†}a+\hbar g\sigma_{x}\left(a^{†}+a\right),$$

where $a\;(a^{†})$ corresponds to the bosonic annihilation (creation) operator of the resonator mode, $\sigma_{x}$ and $\sigma_{z}$ stand for the Pauli operators describing the two-level system. In addition, Ω, ω, and *g*, correspond to the TLS frequency, resonator frequency, and TLS-resonator coupling strength, respectively.

This model has been considered for several applications in quantum information processing [33,47,48,49,50,51]. The ratio between the coupling strength and the resonator frequency g/ω (ω∼Ω) separates the behavior of the system into different regimes [52,53]. In the strong coupling regime, where the coupling strength is much larger than any decoherence or dephasing rate in the system, and for values $g/\omega ≲10^{-2}$, one can perform the rotating wave approximation (RWA) and the system can be described by the Jaynes-Cummings model [54]. As the ratio g/ω is increased beyond the strong coupling regime, there is a breakdown of the RWA and the system must be described by the full quantum Rabi model. We distinguish two main regimes for the later case, the ultra-strong coupling regime (USC) [30,55,56] where the coupling strength is comparable to the resonator frequency g≲ω, and the deep-strong coupling regime (DSC) [31,57] where the interaction parameter is greater than the relevant frequencies g>ω.

**Figure 1:**
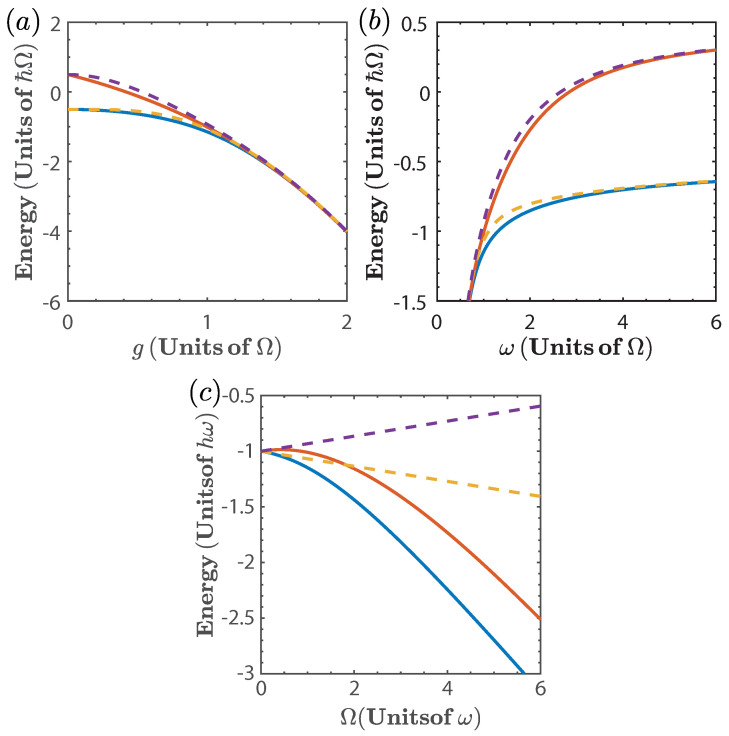
Two lowest energy levels of the quantum Rabi model as a function of (**a**) the coupling strength *g*, with ω=Ω; (**b**) the resonator frequency ω, with g=Ω; and (**c**) the TLS frequency Ω with g=ω. The Solid line denotes the exact diagonalization of Equation (1) and dashed line denotes the approximation given by Equation (2).

In this work we study the quantum cycle for a working substance which is described by the two lowest energy levels of the quantum Rabi model. In order to better describe the behavior of the thermodynamical figures of merit we will use a simple approximation for the first two lowest energy levels, employed on a recent work [5] based on References [58,59]. The approximated energy levels are given by:

(2) $$\begin{array}{cc} E_{0}= & -\hbar \frac{g^{2}}{\omega }-\hbar \frac{\Omega }{2}e^{-2\frac{g^{2}}{\omega^{2}}},\\ E_{1}= & -\hbar \frac{g^{2}}{\omega }+\hbar \frac{\Omega }{2}e^{-2\frac{g^{2}}{\omega^{2}}}, \end{array}$$

where $E_{0}$ and $E_{1}$ refers to the energy of the ground and first excited state, respectively. Figure 1 shows $E_{0}$ and $E_{1}$ as a function of each of the parameters, *g*, ω, and Ω as obtained from Equation (2), compared to their calculation as obtained from the numerical diagonalization of Equation (1). We can see that the approximation given by Equation (2) captures the behavior of the spectrum for all values of *g* and ω considered, while for the case of Ω it is not a good approximation for Ω>ω. Therefore, we will only consider the numerical calculation for the later case.

### 1.2. First Law of Thermodynamic

Let us consider a system with discrete energy levels and whose Hamiltonian $\hat{H}\left(\xi \right)$ depends explicitly on a parameter ξ that can be varied at an arbitrary slow rate. We define the eigenstate and eigenenergies of $\hat{H}\left(\xi \right)$ by $\hat{H}|n;\xi \rangle =E_{n}\left(\xi \right)|n;\xi \rangle$, then, for state $|\psi \rangle =\sum_{n=0}c_{n}|\xi ;n\rangle$, the average energy $\langle E\rangle =\langle \hat{H}\rangle$ of the system takes the form:

(3) $$\langle E\rangle =\sum_{n}p_{n}\left(\xi \right)E_{n}\left(\xi \right).$$

where $p_{n}={\left|c_{n}\right|}^{2}$. The change in the average energy due to an arbitrary quasistatic process involving the modulation of the parameter ξ is given by:

(4) $$\begin{array}{cc} \delta \langle E\rangle = & \sum_{n}E_{n}\left(\xi \right)\frac{\partial }{\partial \xi }p_{n}\left(\xi \right)\delta \xi +\sum_{n}p_{n}\left(\xi \right)\frac{\partial }{\partial \xi }E_{n}\left(\xi \right)\delta \xi \\ = & \delta Q+\delta W. \end{array}$$

where:

(5) $$\begin{array}{cc} \delta Q & =\sum_{n}E_{n}\left(\xi \right)\frac{\partial }{\partial \xi }p_{n}\left(\xi \right)\delta \xi ,\\ \delta W & =\sum_{n}p_{n}\left(\xi \right)\frac{\partial }{\partial \xi }E_{n}\left(\xi \right)\delta \xi . \end{array}$$

Equation (4) is cast in a form reminiscent of the first law of thermodynamics, however, the first term of Equation (4) can only be associated with heat when it is possible to define a temperature in the system, as is the case of an interaction with a thermal reservoir in an isochoric process. Since this is not the case for isoenergetic processes, δQ is known as the energy exchange [25,27], while the second term δW can be identified with the work done. That is, the work done corresponds to the change in the eigenenergies $E_{n}\left(\xi \right)$ which is in agreement with the fact that work can only be performed through a change in generalized coordinates of the systems, which in turn gives rise to a change in the eigenenergies.

### 1.3. Cycle of Operation

**Figure 2:**
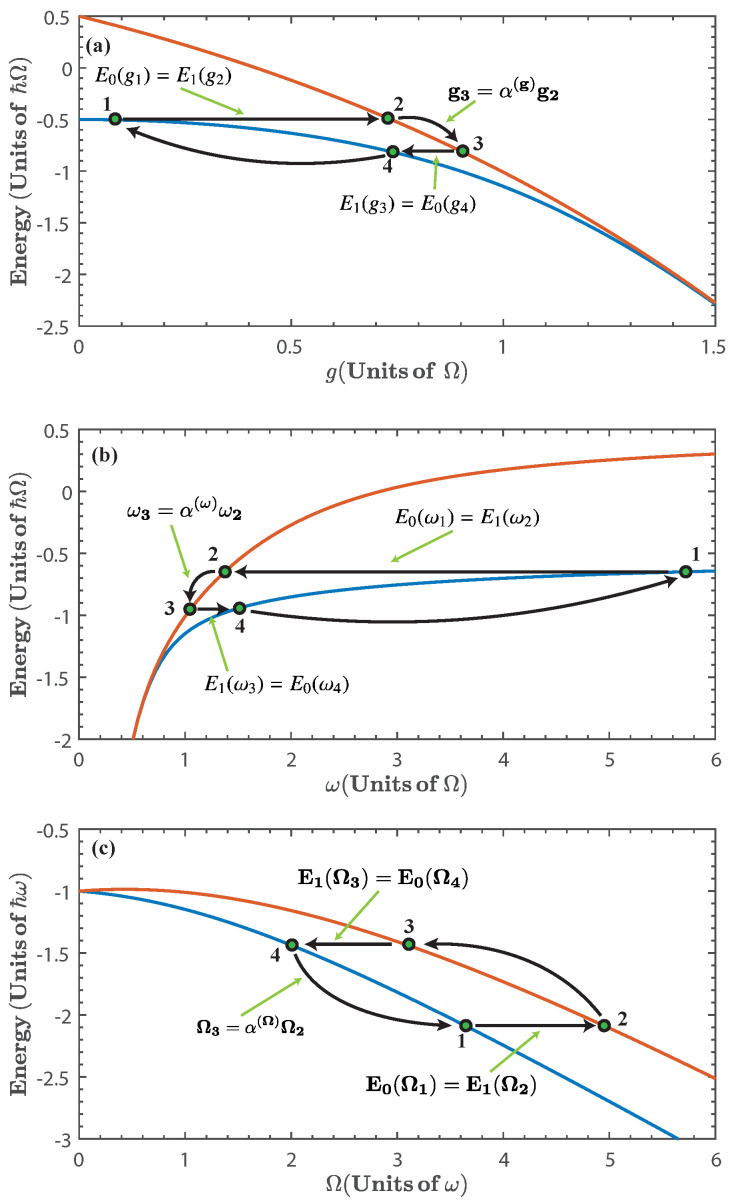
Diagram of the Isoenergetic cycle for (**a**) ξ≡g; (**b**) ξ≡ω; and (**c**) ξ≡Ω. Stages 1→2 and 3→4 correspond to isoenergetic processes, while stages 2→3 and 4→1 correspond to adiabatic processes.

We consider a quantum cycle composed of two quantum adiabatic processes and two isoenergetic ones (see Figure 2). In the quantum adiabatic processes, we change the parameter ξ between two values. This variation must be performed sufficiently slow such that it satisfies the adiabatic approximation [60], which ensures that the populations are kept constant throughout the process. In the isoenergetic process the central idea is to keep constant the initial energy expectation value along the procedure, which means δQ+δW=0. Therefore, both work and energy exchange are nonzero during this process. This means that for ξ∈$\left[\xi_{k},\xi_{\ell }\right]$, we have:

(6) $$\sum_{n}p_{n}\left(\xi_{k}\right)E_{n}\left(\xi_{k}\right)=\sum_{n}p_{n}\left(\xi \right)E_{n}\left(\xi \right)=\sum_{n}p_{n}\left(\xi_{\ell }\right)E_{n}\left(\xi_{\ell }\right),$$

where *k* and *ℓ* refers to the ends points of the compression process (k=1, ℓ=2) or expansion process (k=3, ℓ=4). If we consider that the states at the ends of the isoenergetic process correspond to the ground state and first excited state of the system, as is shown in Figure 2, the processes are termed maximal compression for $E_{0}\left(\xi_{1}\right)=E_{1}\left(\xi_{2}\right)$, and maximal expansion for $E_{1}\left(\xi_{3}\right)=E_{0}\left(\xi_{4}\right)$. These conditions yield $\xi_{2}$ as a function of $\xi_{1}$, and $\xi_{4}$ as a function of $\xi_{3}$; and are referred to as the isoenergetic condition.

For a two-level system, the energy exchange along the isoenergetic process for maximal expansion is given by [19,24]:

(7) $$\begin{array}{c} Q_{\mathrm{in}}^{k\to \ell }=E_{0}\left(\xi_{k}\right)\times \ln\left[|\frac{E_{0}\left(\xi_{\ell }\right)-E_{1}\left(\xi_{\ell }\right)}{E_{0}\left(\xi_{k}\right)-E_{1}\left(\xi_{k}\right)}|\right]+\int_{\xi_{k}}^{\xi_{\ell }}\frac{E_{0}\frac{dE_{1}}{d\xi }-E_{1}\frac{dE_{0}}{d\xi }}{E_{0}\left(\xi \right)-E_{1}\left(\xi \right)}d\xi .\; \end{array}$$

where k=1 and ℓ=2. The isoenergetic process can be modeled as a sequence of steps each composed of an adiabatic processes followed by a driving process on the working substance (see Appendix A), from which expression (7) is recovered. For a maximal compression process we refer to the energy exchange as $Q_{\mathrm{out}}^{k\to \ell }$ (k=3, ℓ=4), and it is obtained by exchanging 0 by 1, and 1 by 0 in Equation (7). The subscripts “in” and “out” denote that energy enters or leaves the system, respectively. We remark that we refer to the quantity $Q_{\mathrm{in}(\mathrm{out})}$ as energy exchange, which is not associated with heat.

In a isoenergetic process there is work performed through the change of the parameter ξ, as can be seen from Equation (5). At the same time, the energy exchange $Q_{\mathrm{in}(\mathrm{out})}^{k\to \ell }$ is supplied by the energy bath in order to keep the expectation value of the Hamiltonian constant. Since in this process the average energy change is zero, we write:

(8) $$Q_{\mathrm{in}(\mathrm{out})}^{k\to \ell }+W_{\mathrm{iso}}^{k\to \ell }=0,$$

where $W_{\mathrm{iso}}^{k\to \ell }$ is the work done by the system. Therefore, we have:

(9) $$W_{\mathrm{iso}}^{k\to \ell }=-Q_{\mathrm{in}(\mathrm{out})}^{k\to \ell }$$

As will be seen in what follows, the isoenergetic processes are the only contribution to the total work extracted.

On the other hand, in a generic adiabatic process the occupation probabilities $p_{n}\left(\xi \right)$ are constant and only work is performed by the system, which is given by [3]:

(10) $$\begin{array}{cc} W_{\left(\mathrm{ad}\right)}^{i\to j} & =\int_{\xi_{i}}^{\xi_{j}}d\xi {\left(\frac{\partial E}{\partial \xi }\right)}_{p_{n}\left(\xi_{i}\right)=p_{n}\left(\xi_{j}\right)=\mathrm{constant}}\\ & =\sum_{n}p_{n}\left(\xi_{i}\right)\left[E_{n}\left(\xi_{j}\right)-E_{n}\left(\xi_{i}\right)\right], \end{array}$$

where the superscripts (i,j) can taken the values (i=2,j=3) for an adiabatic expansion and (i=4,j=1) for the adiabatic compression, respectively. From Figure 2 it is clear that, for each case, the net contribution of the adiabatic processes cancels out, that is, $W_{\left(\mathrm{ad}\right)}^{2\to 3}+W_{\left(\mathrm{ad}\right)}^{4\to 1}=0$. Therefore, the total work extracted is obtained from the isoenergetic processes, which, using Equation (9), can be written as:

(11) $$W_{\mathrm{total}}=W_{\mathrm{iso}}^{1\to 2}+W_{\mathrm{iso}}^{3\to 4}=-Q_{\mathrm{in}}^{1\to 2}-Q_{\mathrm{out}}^{3\to 4}.$$

It is important to notice that within the framework of maximal expansion/compression the system ends in a pure state at the end of each stage. This means that the von Neumann entropy of the system is zero at each process of the cycle, which means that there is no breakdown of the second law for this cycle [14].

Finally, the efficiency of the cycle is:

(12) $$\eta =\frac{W_{\mathrm{total}}}{Q_{\mathrm{in}}}=1-\frac{Q_{\mathrm{out}}^{3\to 4}}{Q_{\mathrm{in}}^{1\to 2}}.$$

It is evident from this expression that to improve the efficiency in the quantum cycle, the ratio $Q_{\mathrm{out}}^{3\to 4}/Q_{\mathrm{in}}^{1\to 2}$ is required to be reduced. As will be shown later, the quantum Rabi system spectrum yields a better minimization of this ratio than most other systems previously considered.

The quantum cycle is specified by the initial parameter $\xi_{1}$ and $\alpha^{\left(\xi \right)}\equiv \xi_{3}/\xi_{2}$, which characterizes the adiabatic process.

The quantum Rabi model depends on three parameters, the coupling strength *g*, the resonator frequency ω, and the TLS frequency Ω. In our cycle, we will fix two of them and vary the third. Furthermore, we will consider the cases of varying each of the three parameters.

We have chosen the first isoenergetic process to be of maximal expansion, which will determine whether ξ should be increased or decreased during the first isoenergetic stage. For the case of ξ=g we must increase the parameter, whereas for ξ=ω and ξ=Ω we must decrease the parameter.

## 2. Quantum Rabi Model as a Working Substance

### 2.1. Case of ξ≡g

Let us start by considering the case of ξ≡g as the parameter to be varied, and fix ω=Ω. This is motivated by experimentally reported control of the coupling strength [38,39,61]. Figure 2a shows the diagram of the quantum cycle corresponding to this case.

**Figure 3:**
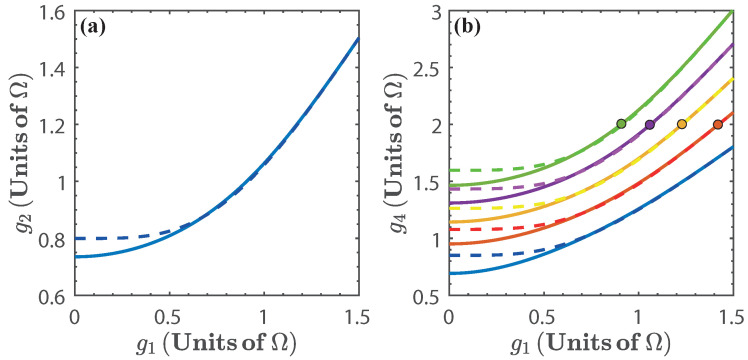
(**a**) $g_{2}$ as a function of $g_{1}$, given by the isoenergetic condition $E_{0}\left(g_{1}\right)=E_{1}\left(g_{2}\right)$; (**b**) $g_{4}$ as a function of $g_{1}$, where $g_{4}$ is obtained from the isoenergetic condition $E_{1}\left(g_{3}\right)=E_{0}\left(g_{4}\right)$, and $g_{3}=\alpha^{\left(g\right)}g_{2}$. We have chosen $\alpha^{\left(g\right)}=1.2$ (blue), $\alpha^{\left(g\right)}=1.4$ (red), $\alpha^{\left(g\right)}=1.6$ (yellow), $\alpha^{\left(g\right)}=1.8$ (purple), and $\alpha^{\left(g\right)}=2$ (green). The dots in Figure 3b indicate the threshold $g_{3}=2\;\Omega$ [57]. Solid lines denote the numerical calculation, and dashed lines are calculated with the approximated energy levels.

Let us first consider the isoenergetic expansion and compression stages. The first isoenergetic process is subject to the isoenergetic condition given by $E_{0}\left(g_{1}\right)=E_{1}\left(g_{2}\right)$ which yields $g_{2}$ as a function of $g_{1}$. This is shown in Figure 3a. Due to the structure of the energy levels, the range of values for $g_{1}$ in which the cycle can be operated is approximately between $0<g_{1}<1.5$. Beyond this value, the energy levels become degenerate and we expect no energy exchange in the isoenergetic process. Therefore, the energy spectrum imposes a bound in the range of values of $g_{1}$ for the operation of the quantum cycle. Similarly, we consider the isoenergetic condition for the compression stage $E_{1}\left(g_{3}\right)=E_{0}\left(g_{4}\right)$ and obtain the values of $g_{4}$ for given $g_{3}$, which is shown in Figure 3b for $\alpha^{\left(g\right)}\in \left[1.2,2\right]$.

From Equation (7), we obtain the energy exchange for the isoenergetic expansion and compression process as:

(13) $$\begin{array}{c} Q_{\mathrm{in}}^{1\to 2}=\frac{2}{\omega^{2}}\left(g_{2}^{2}-g_{1}^{2}\right)\left(\frac{\hbar g_{1}^{2}}{\omega }+\frac{\hbar \Omega }{2}e^{-\frac{2g_{1}^{2}}{\omega^{2}}}\right)-\frac{\hbar }{\omega^{3}}\left(\omega^{2}\left(g_{2}^{2}-g_{1}^{2}\right)+\left(g_{2}^{4}-g_{1}^{4}\right)\right), \end{array}$$

(14) $$\begin{array}{c} Q_{\mathrm{out}}^{3\to 4}=\frac{2}{\omega^{2}}\left(g_{3}^{2}-g_{4}^{2}\right)\left(-\frac{\hbar g_{3}^{2}}{\omega }+\frac{\hbar \Omega }{2}e^{-\frac{2g_{3}^{2}}{\omega^{2}}}\right)+\frac{\hbar }{\omega^{3}}\left(\omega^{2}\left(g_{3}^{2}-g_{4}^{2}\right)+\left(g_{3}^{4}-g_{4}^{4}\right)\right). \end{array}$$

We can see from Equations (13) and (14) that the energy exchange that enters or leaves the system is proportional to $g_{2}^{2}-g_{1}^{2}$ or $g_{3}^{2}-g_{4}^{2}$, respectively. This is to be expected, since the energy exchange between the system and the energy reservoir should depend on how large is the variation of the parameter during the isoenergetic process. Then, by inspecting Figure 2a, we would expect that $Q_{\mathrm{in}}^{1\to 2}/Q_{\mathrm{out}}^{3\to 4}>1$, and that this ratio should be increased by incrementing $\alpha^{\left(g\right)}$.

On the other hand, for the first and second adiabatic processes the work done is given by $W^{2\to 3}=E_{1}\left(g_{3}\right)-E_{1}\left(g_{2}\right)$ and $W^{4\to 1}=E_{0}\left(g_{1}\right)-E_{0}\left(g_{4}\right)$, respectively. Where $g_{3}=\alpha^{\left(g\right)}g_{2}$ and $g_{4}$ is specified by the second isoenergetic condition.

**Figure 4:**
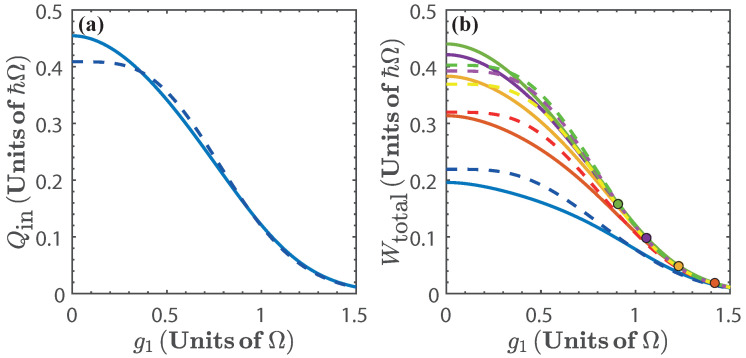
(**a**) Energy exchange $Q_{\mathrm{in}}$ and (**b**) total work extracted, $W_{\mathrm{total}}$, as a function of $g_{1}$, for $\alpha^{\left(g\right)}=1.2$ (blue), $\alpha^{\left(g\right)}=1.4$ (red), $\alpha^{\left(g\right)}=1.6$ (yellow), $\alpha^{\left(g\right)}=1.8$ (purple), and $\alpha^{\left(g\right)}=2$ (green). The dots in Figure 4b indicate the threshold $g_{3}=2\;\Omega$ [57]. Solid lines denote the numerical calculation, and dashed lines are calculated with the approximated energy levels.

Figure 4a shows the energy exchange of the first isoenergetic process as a function of $g_{1}$, as expected, the maximum values are achieved when the isoenergetic condition maximizes the difference $g_{2}^{2}-g_{1}^{2}$, which can also be seen in Figure 2a. The total work extracted, $W_{\mathrm{total}}$, depends on $g_{1}$ and $\alpha^{\left(g\right)}$, as is shown in Figure 4b. Since the total work extracted is obtained from the sum of $Q_{\mathrm{in}}^{1\to 2}$ and $Q_{\mathrm{out}}^{3\to 4}$, the maximum work extracted is found where $Q_{\mathrm{in}}^{1\to 2}$ is maximum and $Q_{\mathrm{out}}^{3\to 4}$ is minimum, that is, at small values of $g_{1}$ and high values of $\alpha^{\left(g\right)}$. In the same way, from Figure 2a we can expect for the total work extracted to vanish as $g_{1}\to 1.5\Omega$. This is a consequence of the energy levels becoming degenerate, and therefore, the isoenergetic process requires almost no variation of *g*, which minimizes both $Q_{\mathrm{in}}^{1\to 2}$ and $Q_{\mathrm{out}}^{3\to 4}$.

**Figure 5:**
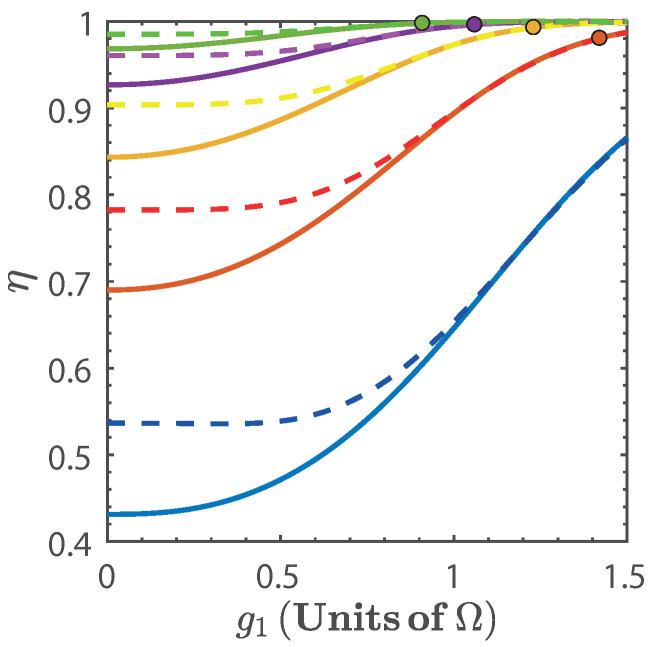
Efficiency η as function $g_{1}$ for $\alpha^{\left(g\right)}=1.2$ (blue), $\alpha^{\left(g\right)}=1.4$ (red), $\alpha^{\left(g\right)}=1.6$ (yellow), $\alpha^{\left(g\right)}=1.8$ (purple), and $\alpha^{\left(g\right)}=2$ (green). The dots indicate the threshold $g_{3}=2\;\Omega$ [57]. In both figures solid line denotes the exact numerical calculation, and dashed line is calculated with the approximated energy levels.

Figure 5 shows the efficiency, η, of the cycle as a function of $g_{1}$ for different values $\alpha^{\left(g\right)}$. From this figure, we see that the efficiency increases with $g_{1}$ as well as with $\alpha^{\left(g\right)}$. This is a consequence of the nonlinear dependence of the energy spectrum on the parameter *g*. Additionally, we see that for finite values of $g_{1}$ the efficiency quickly approaches its maximal theoretical value, instead of asymptotically converging to it [19,24,25]. This can be understood from Figure 2a and Figure 3, since, as $g_{1}$ and $\alpha^{\left(g\right)}$ increase, we can expect that the ratio $Q_{\mathrm{out}}^{3\to 4}/Q_{\mathrm{in}}^{1\to 2}$ to be minimized. This is because the nonlinearity of the energy spectrum with respect to *g* is such that the second isoenergetic process happens closer to the region where the energy levels become degenerate, and from Equation (14) we see that if $g_{4}\to g_{3}$, then $Q_{\mathrm{out}}^{3\to 4}\to 0$. However, this will happen for $W_{\mathrm{total}}\to 0$, as can be seen from Figure 4b. On the other hand, in the region of maximal total work extracted we find values of the efficiency that range in 0.5<η<0.95, depending on the values of $\alpha^{\left(g\right)}$.

### 2.2. Case of ξ≡ω

Now, we consider the choice of ξ≡ω as the parameter to be varied, and fix g=Ω. This is motivated by experimentally reported control of the resonator frequency [40,41,62].

**Figure 6:**
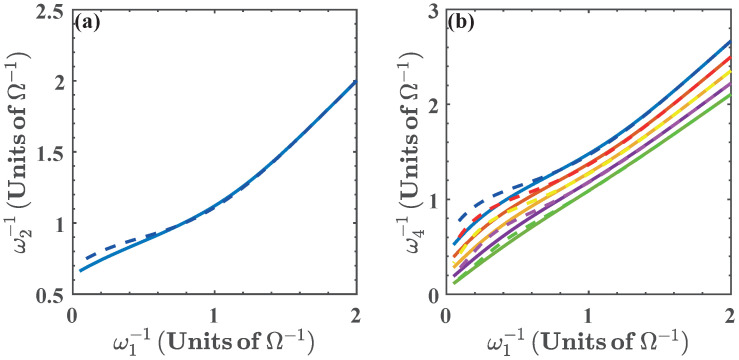
(**a**) $\omega_{2}^{-1}$ as a function of $\omega_{1}^{-1}$ given by the isoenergetic condition $E_{0}\left(\omega_{1}\right)=E_{1}\left(\omega_{2}\right)$; (**b**) $\omega_{4}^{-1}$ as a function of $\omega_{1}^{-1}$, where $\omega_{4}$ is obtained from the isoenergetic condition $E_{1}\left(\omega_{3}\right)=E_{0}\left(\omega_{4}\right)$, and $\omega_{3}=\alpha^{\left(\omega \right)}\omega_{2}$. We have chosen $\alpha^{\left(\omega \right)}=0.75$ (blue), $\alpha^{\left(\omega \right)}=0.80$ (red), $\alpha^{\left(\omega \right)}=0.85$ (yellow), $\alpha^{\left(\omega \right)}=0.90$ (purple), and $\alpha^{\left(\omega \right)}=0.95$ (green). Solid lines denote the numerical calculation, and dashed lines are calculated with the approximated energy levels.

**Figure 7:**
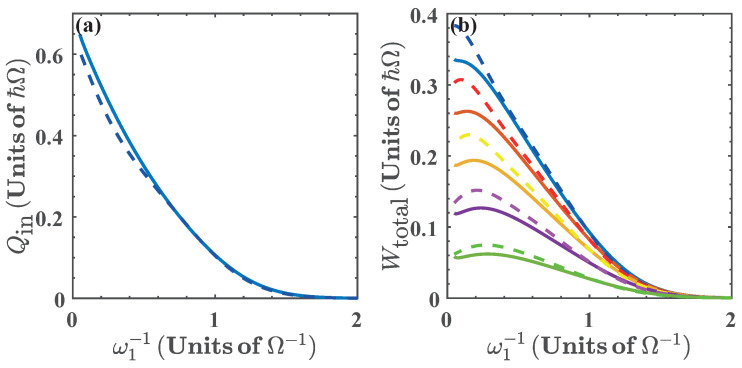
(**a**) Energy exchange $Q_{\mathrm{in}}$ and (**b**) total work extracted $\left(W_{\mathrm{total}}\right)$ as a function of $\omega_{1}^{-1}$ for $\alpha^{\left(\omega \right)}=0.75$ (blue), $\alpha^{\left(\omega \right)}=0.8$ (red), $\alpha^{\left(\omega \right)}=0.85$ (yellow), $\alpha^{\left(\omega \right)}=0.90$ (purple), and $\alpha^{\left(\omega \right)}=0.95$ (green). Solid lines denote the numerical calculation, and dashed lines are calculated with the approximated energy levels.

In this case, the energy exchange for maximal expansion and compression are given by:

(15) $$\begin{array}{c} Q_{\mathrm{in}}^{1\to 2}=\left(-2g^{2}\left(\frac{1}{\omega_{2}^{2}}-\frac{1}{\omega_{1}^{2}}\right)\right)E_{1}\left(\omega_{1}\right)-\frac{4}{3}\hbar g^{4}\left(\frac{1}{\omega_{2}^{3}}-\frac{1}{\omega_{1}^{3}}\right)-\hbar g^{2}\left(\frac{1}{\omega_{2}}-\frac{1}{\omega_{1}}\right), \end{array}$$

(16) $$\begin{array}{c} Q_{\mathrm{out}}^{3\to 4}=\left(2g^{2}\left(\frac{1}{\omega_{3}^{2}}-\frac{1}{\omega_{4}^{2}}\right)\right)E_{2}\left(\omega_{3}\right)+\frac{4}{3}\hbar g^{4}\left(\frac{1}{\omega_{3}^{3}}-\frac{1}{\omega_{4}^{3}}\right)+\hbar g^{2}\left(\frac{1}{\omega_{3}}-\frac{1}{\omega_{4}}\right). \end{array}$$

where $\omega_{2}$, and $\omega_{4}$ are obtained from the isoenergetic conditions $E_{1}\left(\omega_{2}\right)=E_{0}\left(\omega_{1}\right)$ and $E_{0}\left(\omega_{4}\right)=E_{1}\left(\omega_{3}\right)$, respectively. This is presented in Figure 6, for $\alpha^{\left(\omega \right)}\in \left[0.75,0.95\right]$. In the same way as in the previous case, we see that the energy exchange in the isoenergetic processes depends on the amplitude of the variation of the parameter required by the isoenergetic condition. Then, we can expect that the nonlinear dependence of the energy spectrum on ξ=ω will play a similar role as with ξ=g. In what follows we find it convenient to express the results in terms of $1/\omega_{1}$. In this case, the range of values of ω for the operation of the quantum cycle is lower bounded by ω=0.5Ω. Below this value the energy levels become degenerate and there is no total work extracted nor energy exchange as can be seen from Figure 7.

For the first and second adiabatic processes the work done is given by $W^{2\to 3}=E_{1}\left(\omega_{3}\right)-E_{1}\left(\omega_{2}\right)$ and $W^{4\to 1}=E_{0}\left(\omega_{1}\right)-E_{0}\left(\omega_{4}\right)$, respectively. Where $\omega_{3}=\alpha^{\left(\omega \right)}\omega_{2}$, and $\omega_{4}$ is specified by the second isoenergetic process.

The total work extracted, $W_{\mathrm{total}}$, is shown in Figure 7b as a function of $\omega_{1}^{-1}$. We see that for $0.35≲\omega_{1}^{-1}≲0.45$ (in units of $\Omega^{-1}$) we obtain the region of maximal $W_{\mathrm{total}}$ for different values of $\alpha^{\left(\omega \right)}$. This is because in this region, the isoenergetic process requires a large variation of the parameter ω, as can be seen in Figure 2b, which in turn maximizes both $Q_{\mathrm{in}}^{1\to 2}$ and $W_{\mathrm{total}}$. In addition, in Figure 7, we see that as $\omega_{1}^{-1}\to 2\;\Omega^{-1}$, then, $Q_{\mathrm{in}}^{1\to 2}\to 0$ and $W_{\mathrm{total}}\to 0$, which is a consequence of the energy levels becoming degenerate beyond this value of resonator frequency.

**Figure 8:**
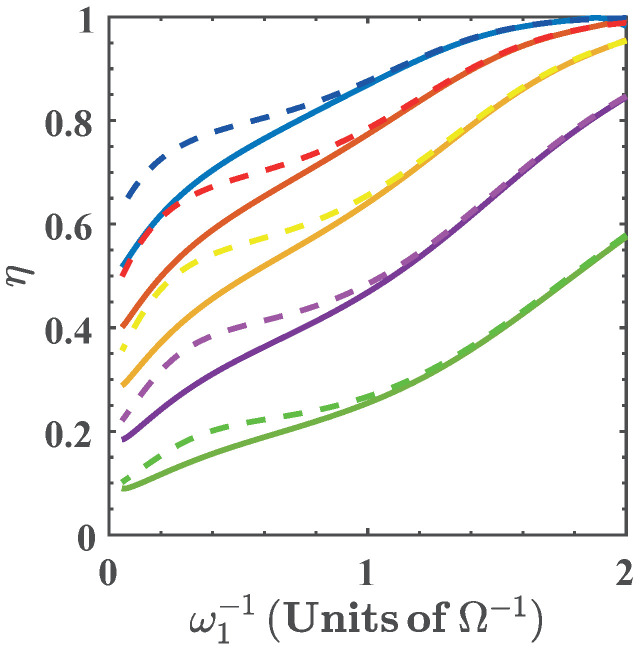
Efficiency as function $\omega_{1}^{-1}$ for $\alpha^{\left(\omega \right)}=0.75$ (blue), $\alpha^{\left(\omega \right)}=0.8$ (red), $\alpha^{\left(\omega \right)}=0.85$ (yellow), $\alpha^{\left(\omega \right)}=0.90$ (purple), and $\alpha^{\left(\omega \right)}=0.95$ (green). Solid lines denote the numerical calculation, and dashed lines are calculated with the approximated energy levels.

In Figure 8 we show the efficiency as a function of $\omega_{1}^{-1}$ for different values of $\alpha^{\left(\omega \right)}$, where we see that the efficiency increases as $\alpha^{\left(\omega \right)}$ is reduced. Notice that the efficiency approaches its maximal theoretical value within the range of $\omega_{1}$ considered. The reason for this is similar to the case of ξ=g, where degeneracy and nonlinearity of the energy spectrum with respect to ω lead to a minimization of the ratio $Q_{\mathrm{out}}^{3\to 4}/Q_{\mathrm{in}}^{1\to 2}$. This can be seen in Figure 2b. At the same time, the maximization of the efficiency occurs as the energy exchange and total work extracted goes to zero. On the other hand, in the region of maximal total work extracted we find values of the efficiency that range in 0.1<η<0.65, depending on the values of $\alpha^{\left(\omega \right)}$.

In both the ξ≡g case and the ξ≡ω case, the nonlinearity and degeneracy of the energy spectrum allows close approach to maximal efficiency of the quantum cycle.

### 2.3. Case of ξ≡Ω

For the final case, we consider the choice ξ≡Ω as the parameter to be varied, and fix g=ω. This is motivated by experimentally reported control of the TLS frequency [42,43]. Since the approximation of Equation (2) breaks down for Ω>ω, we will only consider numerical calculations of the figures of merit.

**Figure 9:**
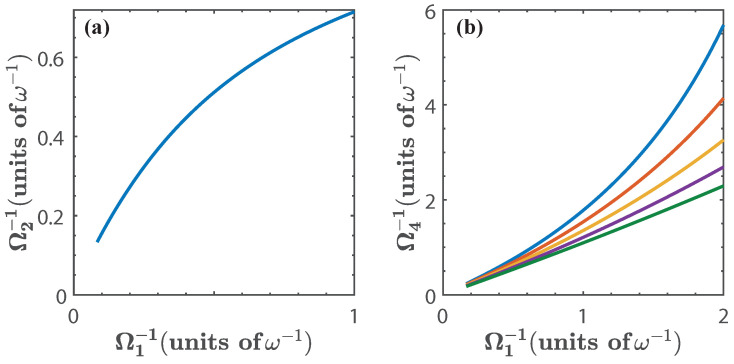
(**a**) shows $\Omega_{2}^{-1}$ as a function of $\Omega_{1}^{-1}$ given by the isoenergetic condition $E_{0}\left(\Omega_{1}\right)=E_{1}\left(\Omega_{2}\right)$; (**b**) shows $\Omega_{4}^{-1}$ as a function of $\Omega_{1}^{-1}$ where $\Omega_{4}$ is obtained from the isoenergetic condition $E_{1}\left(\Omega_{3}\right)=E_{0}\left(\Omega_{4}\right)$, and $\Omega_{3}=\alpha^{\left(\Omega \right)}\Omega_{2}$. We have chosen $\alpha^{\left(\Omega \right)}=0.75$ (blue), $\alpha^{\left(\Omega \right)}=0.8$ (red), $\alpha^{\left(\Omega \right)}=0.85$ (yellow), $\alpha^{\left(\Omega \right)}=0.90$ (purple), and $\alpha^{\left(\Omega \right)}=0.95$ (green). In this case we have only considered the exact numerical calculation.

The solution for the isoenergetic condition is shown in Figure 9, for $\alpha^{\left(\Omega \right)}\in \left[0.75,0.95\right]$. We see that this case differs from the previous ones in that there is no need to limit the parameter Ω to a specific range of values because there is no degeneracy of the energy levels. Nonetheless, we have restricted the values of Ω to the range 0.5<Ω<6 (in units of ω) to facilitate the comparison with the other cases.

**Figure 10:**
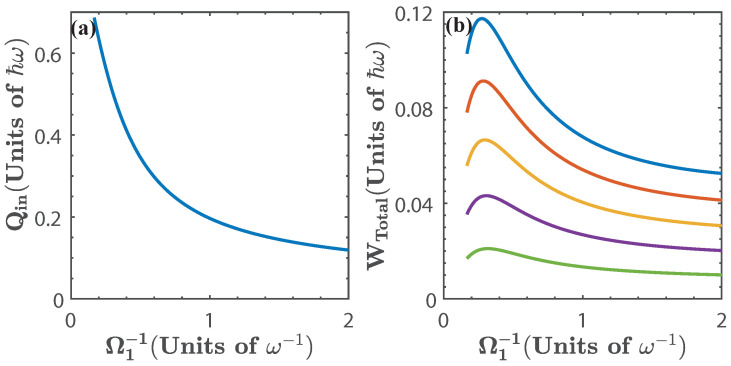
(**a**) Energy exchange $Q_{\mathrm{in}}$ and (**b**) total work extracted $\left(W_{\mathrm{total}}\right)$ as a function of $\Omega_{1}^{-1}$ for $\alpha^{\left(\Omega \right)}=0.75$ (blue), $\alpha^{\left(\Omega \right)}=0.8$ (red), $\alpha^{\left(\Omega \right)}=0.85$ (yellow), $\alpha^{\left(\Omega \right)}=0.90$ (purple), and $\alpha^{\left(\Omega \right)}=0.95$ (green).

The total work extracted is shown in Figure 10, it can be seen that it is considerably smaller than in previous cases, as expected from inspecting the energy spectrum in Figure 2c. Since in this case there is no degeneracy, the total work extracted does not vanish within the chosen range of the parameter.

**Figure 11:**
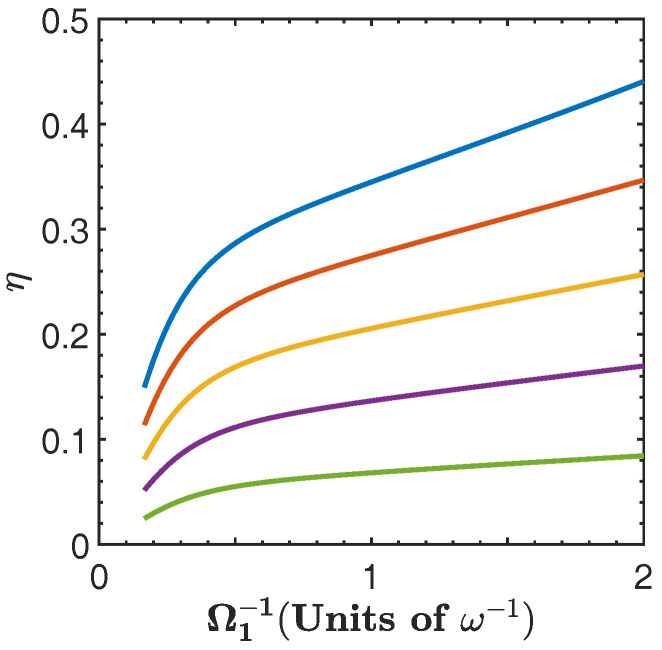
Efficiency as a function of $\Omega_{1}^{-1}$ for different values of $\alpha^{\left(\Omega \right)}$ given by $\alpha^{\left(\Omega \right)}=0.75$ (blue), $\alpha^{\left(\Omega \right)}=0.8$ (red), $\alpha^{\left(\Omega \right)}=0.85$ (yellow), $\alpha^{\left(\Omega \right)}=0.90$ (purple), and $\alpha^{\left(\Omega \right)}=0.95$ (green).

In Figure 11 we show the efficiency as a function of $\Omega_{1}^{-1}$ for different values of $\alpha^{\left(\Omega \right)}$. Here, the efficiency is smaller than those in the previous cases. This is because the functional dependence of the energy levels on Ω is closer to linear behavior as compared with the other two parameters that were previously considered.

Finally, we briefly discuss a possible implementation of this cycle in superconducting circuits. As we mentioned before, the operation of the cycle under consideration relies on varying some parameter of the system at each step. The modulation of the parameters considered in the manuscript has been demonstrated experimentally in superconducting circuit realizations [38,39,40,41,42,43,61,62]. This allows the implementation of the adiabatic stages by a slow variation of the parameters. On the other hand, the isoenergetic processes could be engineered by the method shown in the appendix, as a sequence of steps each composed of an adiabatic processes followed by a driving process. The implementation of drivings is straightforward, which means that the possibility of varying the system parameters in superconducting circuit realizations also enable the implementation of the isoenergetic processes.

## 3. Conclusions

We have studied the performance of an quantum cycle with a working substance described by the quantum Rabi model. We have considered the variation of each of the parameters of the system, *g*, ω and Ω. We use a simple approximation of the energy levels, which helps to understand the behavior of the figures of merit.

We find that the nonlinear dependence of the energy levels on either the coupling strength, *g*, or the resonator frequency, ω, allows for the cycle efficiency to closely approach the maximum. This occurs when the parameter is sufficiently increased (for *g*) or decreased (for ω) in the first adiabatic stage. On the other hand, maximal total work extracted is found at efficiencies in the range of 0.5<η<0.95 for the variation of *g*, and in the range of 0.1<η<0.65 for the variation of ω, which depend on the changes induced by the adiabatic processes.

Finally, we considered the case of varying the TLS frequency Ω. We find that the total work extracted and the efficiency are considerably smaller than those in the previous cases. This is because the functional dependence of the energy levels with Ω is closer to linear behavior as compared with the other two parameters.

Summarizing, the degeneracy and nonlinearity of the energy spectrum of the working substance play the role of enhancing the performance of the quantum cycle. These results may encourage the consideration of these properties of the energy spectrum to optimize the performance of a quantum cycle composed of adiabatic and isoenergetic processes in future studies. 

## Appendix A. Simulation of Isoenergetic Process

In this appendix we show that expression (7) for the energy exchange between the system and the *energy reservoir* can be reproduced by regarding the isoenergetic process as a sequence of steps each composed of an infinitesimal adiabatic change of the parameter ξ followed by a driving process, simulating the action of the energy reservoir. While this is exact, it is not possible to implement an infinitesimal adiabatic change, nonetheless, an isoenergetic process could be approximated by a finite sequence of steps with arbitrary precision, by considering at each step an arbitrarily small adiabatic process, that is followed by a driving process. In this case, both actions of each step could be implemented with current technology in superconducting circuits.

Let us consider a system described by a Hamiltonian that depends on a parameter ξ, it means:

(A1) $$H\left(\xi \right)=\sum_{j}\epsilon_{j}\left(\xi \right)|\phi_{j}\left(\xi \right)\rangle \langle \phi_{j}\left(\xi \right)|$$

with $\epsilon_{j}\left(\xi \right)$ and $|\phi_{j}\left(\xi \right)\rangle$ are the *j*-th eigenenergy and eigenstate, respectively.

Now, we consider an isoenergetic change in the parameter ξ, with energy expectation value $E_{o}\leq \epsilon_{1}\left(\xi \right)$. In this case, we can write the quantum state of the system as a linear superposition of the two lowest levels. Then in an arbitrary point $\xi =\xi_{a}$ of the isoenergetic process the state of the system reads:

(A2) $$|\psi \left(\xi_{a}\right)\rangle =\sqrt{p(\xi_{a})}|\phi_{0}(\xi_{a}\rangle +\sqrt{1-p(\xi_{a})}|\phi_{1}\left(\xi_{a}\right)\rangle$$

and its energy is given by:

(A3) $$E_{o}=p\left(\xi_{a}\right)\left[\epsilon_{0}\left(\xi_{a}\right)-\epsilon_{1}\left(\xi_{a}\right)\right]+\epsilon_{1}\left(\xi_{a}\right),$$

then:

(A4) $$p\left(\xi_{a}\right)=\frac{E_{o}-\epsilon_{1}\left(\xi_{a}\right)}{\epsilon_{0}\left(\xi_{a}\right)-\epsilon_{1}\left(\xi_{a}\right)}.$$

We simulate an infinitesimal isoenergetic change by an adiabatic change in the parameter ξ and a driving in order to keep the energy constant. Then, first we change adiabatically $\xi_{a}\to \xi_{a}+\delta \xi$, obtaining the state:

(A5) $$|\bar{\psi }\left(\xi_{a}+\delta \xi \right)\rangle =\sqrt{p(\xi_{a})}|\phi_{0}\left(\xi_{a}+\delta \xi \right)\rangle +\sqrt{1-p(\xi_{a})}|\phi_{1}\left(\xi_{a}+\delta \xi \right)\rangle$$

with energy:

(A6) $$\bar{E}=p\left(\xi_{a}\right)\left[\epsilon_{0}\left(\xi_{a}+\delta \xi \right)-\epsilon_{1}\left(\xi_{a}+\delta \xi \right)\right]+\epsilon_{1}\left(\xi_{a}+\delta \xi \right).$$

Performing a driving process in the transition 0–1 of the system we obtain the state:

(A7) $$|\psi \left(\xi_{a}+\delta \xi \right)\rangle =\sqrt{p(\xi_{a}+\delta \xi )}|\phi_{0}\left(\xi_{a}+\delta \xi \right)\rangle +\sqrt{1-p(\xi_{a}+\delta \xi )}|\phi_{1}\left(\xi_{a}+\delta \xi \right)\rangle$$

with energy $E_{o}$. In order to keep the energy constant, there must be an energy flow from the driving machine to the system given by $\delta E=E_{o}-\bar{E}$ given by:

(A8) $$\begin{array}{ccc} \delta E & = & p\left(\xi_{a}\right)\left[\left(\epsilon_{0}\left(\xi_{a}\right)-\epsilon_{0}\left(\xi_{a}+\delta \xi \right)\right)-\left(\epsilon_{1}\left(\xi_{a}\right)-\epsilon_{1}\left(\xi_{a}+\delta \xi \right)\right)\right]+\left(\epsilon_{1}\left(\xi_{a}\right)-\epsilon_{1}\left(\xi_{a}+\delta \xi \right)\right)\\ & = & \left[\frac{E_{o}-\epsilon_{1}\left(\xi_{a}\right)}{\epsilon_{0}\left(\xi_{a}\right)-\epsilon_{1}\left(\xi_{a}\right)}\right]\left[\left(\epsilon_{0}\left(\xi_{a}\right)-\epsilon_{0}\left(\xi_{a}+\delta \xi \right)\right)-\left(\epsilon_{1}\left(\xi_{a}\right)-\epsilon_{1}\left(\xi_{a}+\delta \xi \right)\right)\right]+\left(\epsilon_{1}\left(\xi_{a}\right)-\epsilon_{1}\left(\xi_{a}+\delta \xi \right)\right) \end{array}$$

As δξ is a small change, the eigenenergies in the point $\xi =\xi_{a}+\delta \xi$ reads:

(A9) $$\begin{array}{c} \epsilon_{0}\left(\xi_{a}+\delta \xi \right)\approx \epsilon_{0}\left(\xi_{a}\right)+\left(\frac{d}{d\xi }\epsilon_{0}\left(\xi \right)\right){|}_{\xi =\xi_{a}}\delta \xi \\ \epsilon_{1}\left(\xi_{a}+\delta \xi \right)\approx \epsilon_{1}\left(\xi_{a}\right)+\left(\frac{d}{d\xi }\epsilon_{1}\left(\xi \right)\right){|}_{\xi =\xi_{a}}\delta \xi \end{array}$$

then we obtain for δE:

(A10) $$\begin{array}{cc} \delta E= & -\left[\frac{E_{o}-\epsilon_{1}\left(\xi_{a}\right)}{\epsilon_{0}\left(\xi_{a}\right)-\epsilon_{1}\left(\xi_{a}\right)}\right]\left[\epsilon_{0}^{\prime }\left(\xi_{a}\right)-\epsilon_{1}^{\prime }\left(\xi_{a}\right)\right]\delta \xi -\epsilon_{1}^{\prime }\left(\xi \right)\delta \xi \\ \Rightarrow \delta E= & -\left[\frac{E_{o}}{\epsilon_{0}\left(\xi_{a}\right)-\epsilon_{1}\left(\xi_{a}\right)}\right]\left[\epsilon_{0}^{\prime }\left(\xi_{a}\right)-\epsilon_{1}^{\prime }\left(\xi_{a}\right)\right]\delta \xi +\left[\frac{\epsilon_{1}\left(\xi_{a}\right)\epsilon_{0}^{\prime }\left(\xi_{a}\right)-\epsilon_{0}\left(\xi_{a}\right)\epsilon_{1}^{\prime }\left(\xi_{a}\right)}{\epsilon_{0}\left(\xi_{a}\right)-\epsilon_{1}\left(\xi_{a}\right)}\right]\delta \xi . \end{array}$$

If the change δξ is infinitesimal, then δξ→dξ, δE→dE, and the total energy flow from the driving machine to the system along the isoenergetic process reads:

(A11) $$\begin{array}{ccc} \Delta E=\int_{\xi_{\mathrm{initial}}}^{\xi_{\mathrm{final}}}dE & = & -E_{o}\int_{\xi_{\mathrm{initial}}}^{\xi_{\mathrm{final}}}\frac{\epsilon_{0}^{\prime }\left(\xi \right)-\epsilon_{1}^{\prime }\left(\xi \right)}{\epsilon_{0}\left(\xi \right)-\epsilon_{1}\left(\xi \right)}d\xi +\int_{\xi_{\mathrm{initial}}}^{\xi_{\mathrm{final}}}\frac{\epsilon_{1}\left(\xi \right)\epsilon_{0}^{\prime }\left(\xi \right)-\epsilon_{0}\left(\xi \right)\epsilon_{1}^{\prime }\left(\xi \right)}{\epsilon_{0}\left(\xi \right)-\epsilon_{1}\left(\xi \right)}d\xi \;\\ & = & -E_{o}\ln\left[\frac{\epsilon_{0}\left(\xi_{\mathrm{final}}\right)-\epsilon_{1}\left(\xi_{\mathrm{final}}\right)}{\epsilon_{0}\left(\xi_{\mathrm{initial}}\right)-\epsilon_{1}\left(\xi_{\mathrm{initial}}\right)}\right]+\int_{\xi_{\mathrm{initial}}}^{\xi_{\mathrm{final}}}\frac{\epsilon_{1}\left(\xi \right)\epsilon_{0}^{\prime }\left(\xi \right)-\epsilon_{0}\left(\xi \right)\epsilon_{1}^{\prime }\left(\xi \right)}{\epsilon_{0}\left(\xi \right)-\epsilon_{1}\left(\xi \right)}d\xi \end{array}$$

which corresponds to the expression of the energy exchange of Equation (7) considered in our quantum cycle. Therefore, we can consider a physical implementation of an approximate isoenergetic process as a sequence of steps, each composed by an arbitrarily small adiabatic change in the parameter (δξ) followed by a driving to restore the energy, where the latter plays the role of the *energy reservoir*. Both of these processes are available in superconducting circuit realizations.
